# The spent culture supernatant of *Pseudomonas syringae* contains azelaic acid

**DOI:** 10.1186/s12866-018-1352-z

**Published:** 2018-11-28

**Authors:** Sree Gowrinadh Javvadi, Paola Cescutti, Roberto Rizzo, Valentina Lonzarich, Luciano Navarini, Danilo Licastro, Corrado Guarnaccia, Vittorio Venturi

**Affiliations:** 1 International Centre for Genetic Engineering and Biotechnology, Trieste, Italy; 2 Department of Life Sciences, University of Trieste, Trieste, Italy; 3 Illycaffè S.p.a, Trieste, Italy; 4 CBM S.c.r.l., Area Science Park-Basovizza, Trieste, Italy

**Keywords:** Quorum sensing, *Pseudomonas syringae* pv*. actinidiae*, Metabolomics, Azelaic acid

## Abstract

**Background:**

*Pseudomonas syringae* pv*. actinidiae* (PSA) is an emerging kiwifruit bacterial pathogen which since 2008 has caused considerable losses. No quorum sensing (QS) signaling molecule has yet been reported from PSA and the aim of this study was to identify possible intercellular signals produced by PSA.

**Results:**

A secreted metabolome analysis resulted in the identification of 83 putative compounds, one of them was the nine carbon saturated dicarboxylic acid called azelaic acid. Azelaic acid, which is a nine-carbon (C9) saturated dicarboxylic acid, has been reported in plants as a mobile signal that primes systemic defenses. In addition, its structure,(which is associated with fatty acid biosynthesis) is similar to other known bacterial QS signals like the Diffusible Signal Facor (DSF). For these reason it could be acting as s signal molecule. Analytical and structural studies by NMR spectroscopy confirmed that in PSA spent supernatants azelaic acid was present. Quantification studies further revealed that 20 μg/L of were present and was also found in the spent supernatants of several other *P. syringae* pathovars. The RNAseq transcriptome study however did not determine whether azelaic acid could behave as a QS molecule.

**Conclusions:**

This study reports of the possible natural biosynthesis of azelaic acid by bacteria. The production of azelaic acid by *P. syringae* pathovars can be associated with plant-bacteria signaling.

**Electronic supplementary material:**

The online version of this article (10.1186/s12866-018-1352-z) contains supplementary material, which is available to authorized users.

## Background

Bacteria produces and secretes an array of low molecular weight secondary metabolites; in most cases their function remains unknown. Several have been implicated in signaling, as having in vitro antimicrobial activity or being involved in host-pathogen interactions. The production of small low molecular weight microbially produced molecules is also hugely exploited by many private companies around the world [1, 2].

It is now known that bacteria produces and senses a variety of small signals that enable them to act coordinately as a community in a process known as quorum sensing (QS) [3, 4]. In the last 20 years, many bacterial species have been reported to produce QS signals and it is reasonable to assume that most, if not all, use small chemical compounds to communicate with their neighborhood [5–7].

In the *Pseudomonas* group of phytopathogens, QS is a common regulatory mechanism. It occurs commonly via the LuxI/LuxR circuit, which produces and responds to *N*-acylhomoserine lactone (AHL) signals. LuxI-family proteins are the signal synthases that are responsible for producing AHLs, whereas LuxR-family members are the AHL-responsive cytoplasmic DNA-binding transcriptional activators. The LuxR-family protein forms a complex with the AHL at threshold concentrations and affects the transcription of target genes [8–10]. Several Gram-negative bacteria also produce long chain fatty acids and fatty acid methyl esters as QS signal molecules. Some examples of these types of molecules are (i) PQS (pseudomonas quinolone signal) 2-heptyl-3-hydroxy-4(1H)-quinolone is produced by *Pseudomonas aeruginosa*, (ii) HHQ signal, 2-heptyl-4 (1H)-quinolone by *Bacillus atrophaeus* and *Pseudomonas* [11, 12] (iii) the DSF, diffusible signaling factor, cis-11-methyl-2-dodecenoic acid by *Xanthomonas* and *Burkholderia* [13–15] and (iv) 3OH-PAME, hydroxyl-palmitic acid methyl ester by *Ralstonia solanacearum* [16].

QS-dependent regulation in bacteria is most often involved in the coordinated community action of bacteria like inhibiting host defense responses, biofilm formation, production of extracellular enzymes and virulence factors that ultimately leads to the colonization of a niche in the host [17–19]. Interestingly, recent studies on QS have evidenced deviation from the canonical AHL responsive LuxI/R system and its involvement in virulence [20]. For instance, we have reported that *Pseudomonas syringae* pv*. actinidiae* (PSA) is devoid of a LuxI/R canonical QS system and does not produce AHLs. The PSA genome does not possess a LuxI AHL synthase gene. Interestingly however, it possesses three LuxR- proteins closely related to the QS LuxR family (these are called LuxR solos). These LuxR solos were later identified either responding to exogenous AHLs or to a yet unidentified plant low molecular mass compound [21, 22].

PSA is an emerging plant pathogen which causes canker or leaf spot on kiwifruit plants (*Actinidiae* spp.) [23]. PSA was first described in Japan in the 1980s, and later it was reported in Korea and Europe causing damage in kiwifruit orchards [24]. In the European region, the disease was first noticed in central Italy in 1992 where it remained sporadic and with a low incidence for 15 years [25] however in 2007/2008 serious economic losses started to be observed particularly in the Lazio region as well as in the rest of the world [26].

To our knowledge no QS system has been yet been identified in PSA. In the *P. syringae* group of species, only very few pathovars have been reported to possess a complete archetypical AHL QS system [27–29]. In the rest of the pathovars it remains unknown whether they possess a QS system, which is yet to be identified. The purpose of this study was to investigate the production of low molecular weight compounds by PSA that could be involved in QS signaling.

An extracellular metabolome of PSA was determined and among the compounds identified was a nine carbon di-carboxylic acid molecule that could be azelaic acid. It was of interest to investigate this possibility since it has been previously reported as a mobile signal in plants and its structure is related to other known QS molecules. Jung et al. for the first time demonstrated that azelaic acid primes systemic defenses in *Arabidopsis thaliana*. Elevated levels of azelaic acid were detected in the vascular sap upon challenging with the *P. syringae* pathogen suggesting that this molecule can move systemically, most probably through the plant vasculature and contribute to long-distance signaling in plant defense against pathogens [30, 31]. The mechanism of azelaic acid synthesis in plant has however not yet been elucidated and it has recently been postulated that it could be derived from lipids via peroxidation [32].

Azelaic acid is a straight chained nine-carbon (C9) saturated dicarboxylic acid with a molecular weight of 188.22 and a melting point of 105.5 °C. It occurs naturally in whole grain cereals, rye, and barley [33]. We report here that *P. syringae* pathovars could also produce azelaic acid hence be responsible, at least in part, of azelaic acid accumulation in plants. Our analytical chemical studies have confirmed azelaic acid presence in the culture supernatants being a common feature in *Pseudomonas syringae* pathovars. From our studies, we concluded that azelaic acid in PSA is not involved in cell-cell signaling in response to cell density. We report the possible biosynthesis of azelaic acid by *P. syringae* pathovars and possible biological roles are discussed.

## Results

### Metabolome studies of *P. syringae* pv. *actinidiae* and *P. savastanoi* pv. *savastanoi*

With the aim of identifying novel signaling molecules produced by *P. syringae* pv. *actinidiae* (PSA), we analyzed the extracellular metabolome profile using gas chromatography coupled with mass spectrometry (GC-MS). PSA was cultured in M9 liquid growth medium with glucose as the sole carbon source (M9-glucose), cells were then harvested in the late logarithmic/early stationary phase of growth and the spent cell free supernatant was subsequently extracted with ethyl acetate for total metabolome analysis by subjecting it to GC-MS. Chromatograms revealed the putative presence of 83 different metabolites (Additional file 1), among which was the 9-carbon dicarboxylic acid azelaic acid. A similar metabolome experiment was performed using another closely related plant pathogen, namely *P. savastanoi* pv. *savastanoi* (PSV), which causes the olive knot disease in the olive plant [34]. Interestingly, like PSA, the metabolome of PSV also contained small amounts of azelaic acid (Additional file 2). As a control, the same analysis was performed using the M9 glucose medium and in this case, no azelaic acid was detected (Additional file 3).

### Extraction and purification of azelaic acid from PSA spent supernatants

**Fig. 1:**
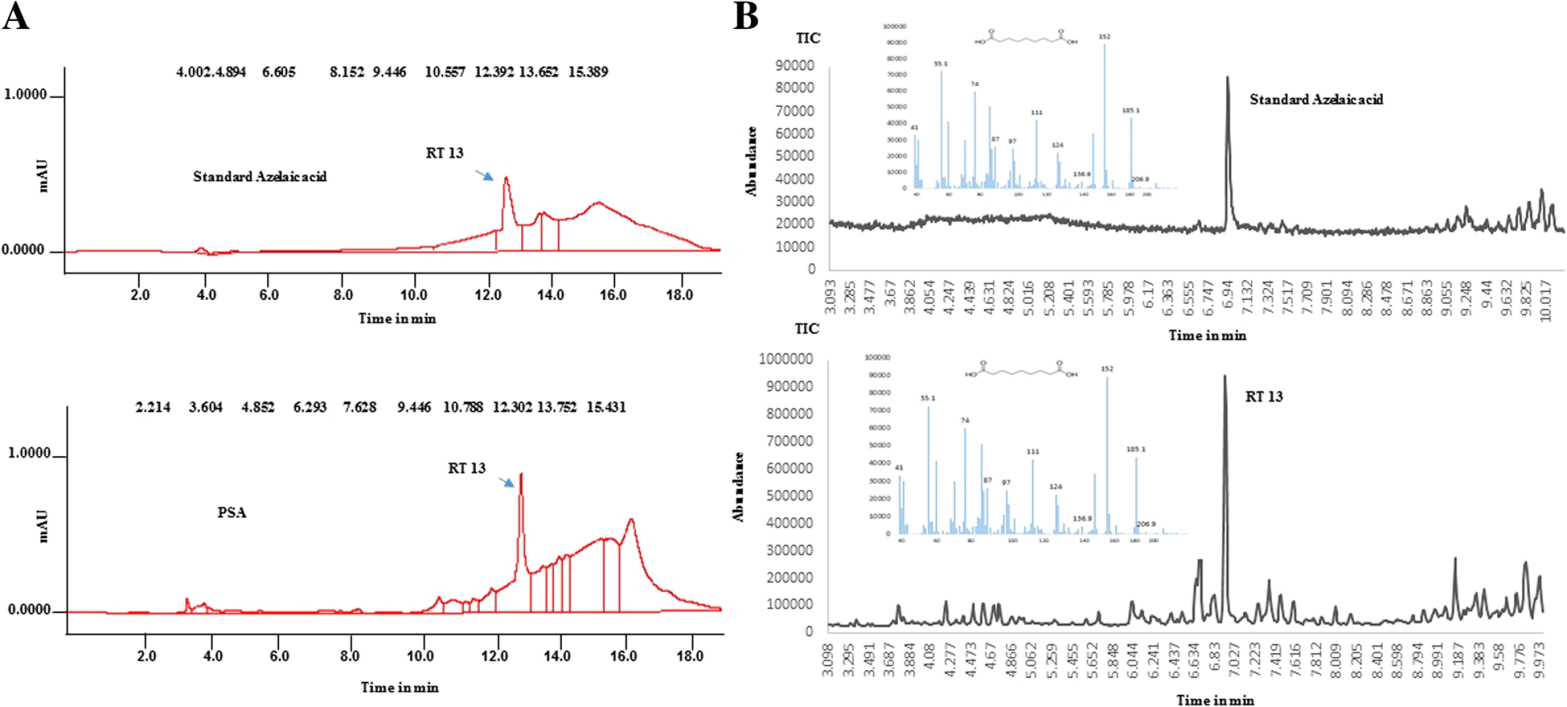
Representative HPLC and GC-MS chromatograms of standard azelaic acid and azelaic acid isolated from PSA. The presence of azelaic acid in the PSA extracellular metabolome was determined in comparison with standard azelaic acid. Using HPLC on C_18_ reverse phase column compounds were analyzed and they collectively retained at similar retention time (RT) 13 min (**a**). GC-MS analysis of HPLC collected fraction at RT 13, showing similar mass fragmentation pattern with standard azelaic acid, confirms the presence of azelaic acid (**b**)

In order to unequivocally confirm the presence of azelaic acid in the PSA supernatants, we purified azelaic acid from PSA spent supernatants by HPLC using a C18 reverse phase column with detection wavelength at 220 nm. HPLC fractions with azelaic acid were analyzed by GC-MS and showed the ion fragments at *m/z* 41, 55.1, 74, 87, 97, 111, 124, 136.9, 152, 185.1, and 206.9 (Fig. 1a, b) with retention time and *m/z* values that were identical to that of standard azelaic acid. Performing the quantification of azelaic acid from the HPLC purified peaks/fractions using LC-MS proved that PSA spent culture supernatants contained approximately 20 μg/L (Additional file 4).

### Structure elucidation of azelaic acid by NMR spectroscopy

**Fig. 2:**
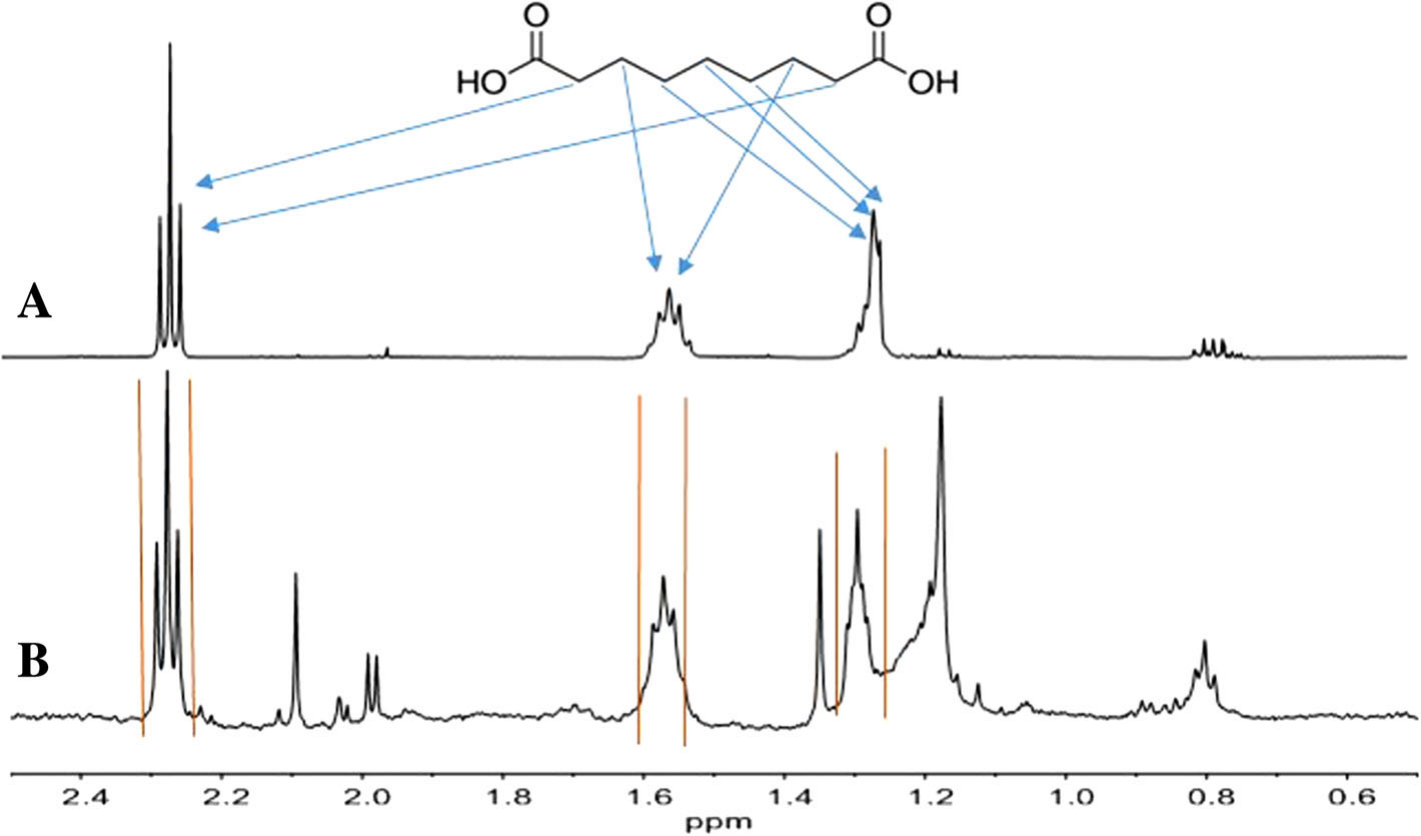
^1^H-NMR studies of Azelaic acid. Spectra of a solution of standard azelaic acid (**a**) and of the azelaic acid extracted and purified from PSA (**b**) recorded at 25 °C in CD_3_Cl

The structure determination of the putative azelaic acid extracted from the PSA spent supernatants, NMR spectroscopy was carried out comparing the extracted sample with a commercially available azelaic acid standard. The ^1^H NMR spectrum of the standard sample in CDCl_3_ is shown in Fig. 2a, with the relative peak assignment. The putative azelaic acid sample, was dissolved in CDCl_3_ and the obtained spectrum was compared with that from the standard. Apart from spurious signals due to PSA extraction contaminants, all the relevant resonance peaks of the standard azelaic acid are present in the spectrum and they are indicated in Fig. 2b between vertical markers. It is also worth noting that the signals of the spectra in Fig. 2a and b exhibit the identical proper multiplicity due to *J* coupling between vicinal hydrogen atoms.

**Fig. 3:**
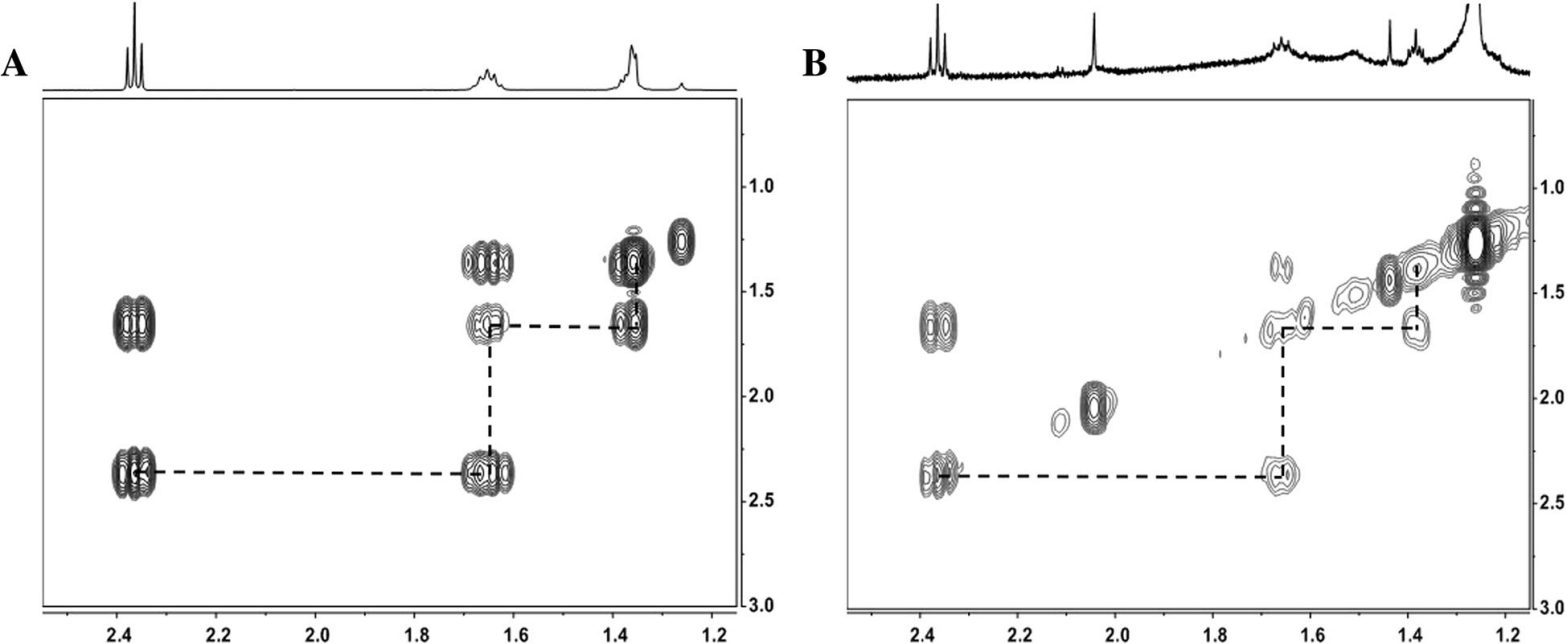
Depicts COSY 2-D spectrum of standard and azelaic acid isolated from PSA. The relevant cross-peaks, connected with dashed lines, are identical in the standard sample and in the extracted sample (**a** and **b**)

To better characterize the PSA extracted azelaic acid, COSY 2D-NMR spectra were obtained for both standard and the extracted samples. The two spectra are shown in Fig. 3a (standard) and Fig. 3b (extracted sample). Again, the relevant cross-peaks, indicating the structural proximity of specific hydrogen atoms connected with dashed lines, are identical in the standard sample and in the extracted sample. In conclusion, both NMR analyses confirmed that the purified compound was azelaic acid.

### Azelaic acid production when PSA is grown in different carbon sources

**Fig. 4:**
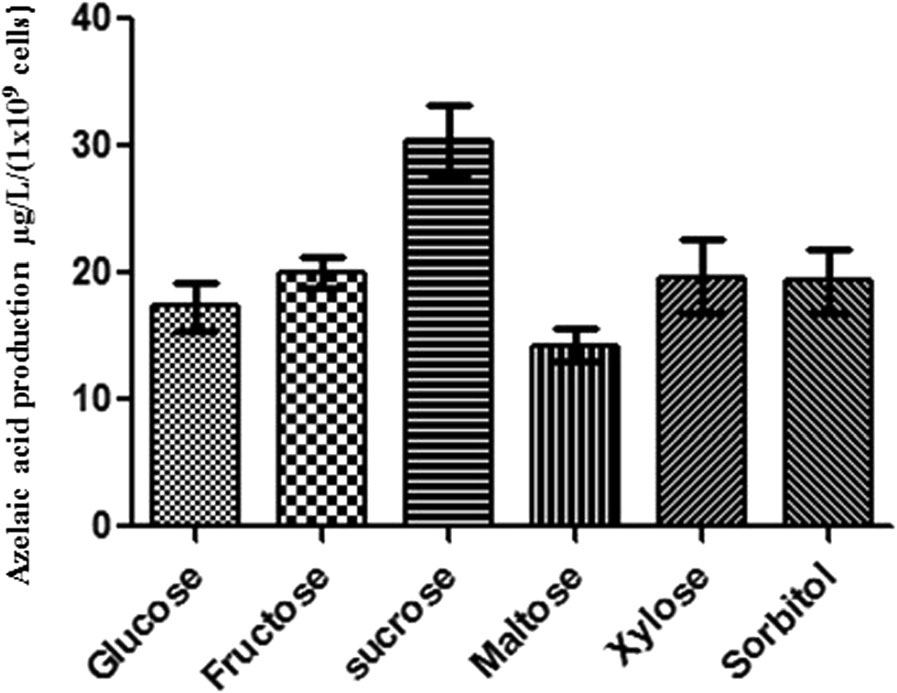
Represents PSA azelaic acid production efficiency in different sugars. Production of azelaic acid by PSA grown in M9 minimal medium with respective sugars as the sole carbon source. Azelaic acid from spent medium was extracted and quantified at 70 h. Bar indicates the means and standard deviation of experiment performed in three biological replicates. Asterisks indicate significant difference between the azelaic acid production in glucose and other carbon sources (*P* < 0.05)

It was of interest to assess the presence of azelaic acid at different phases of the growth of PSA and using different sources of carbon. PSA was cultured in M9 medium replacing glucose with different carbohydrates like fructose, sucrose, maltose, xylose and sorbitol as sole carbon source. Fructose, sucrose, maltose, xylose and sorbitol all supported growth of PSA and resulted in the presence of azelaic acid in spent supernatants. The use of sucrose enhanced the production by 1.6 fold compared to glucose (Fig. 4). Azelaic acid production was then assessed in the different phases of growth. The highest levels of azelaic acid (36 μg/L) accumulated during the mid-exponential phase of growth (Table 1; Additional file 5).

**Table 1:** Represents the detection of azelaic acid at different growth phase

S.no	Time (hr)	OD_600_ _1_st set	OD_600_ 2nd set	Azelaic acid concentration μg/L 1st set	Azelaic acid concentration μg/L 2nd set
1	0	0.04	0.04	0	0
2	24	0.36	0.4	5.2	7.3
3	42	1.2	0.9	36.5	31.1
4	66	1.9	1.4	31.0	27.6
5	72	1.3	1.1	21.2	24.4
6	90	0.9	0.8	19.8	16.6

### *Pseudomonas syringae* pathovars also produce azelaic acid

**Fig. 5:**
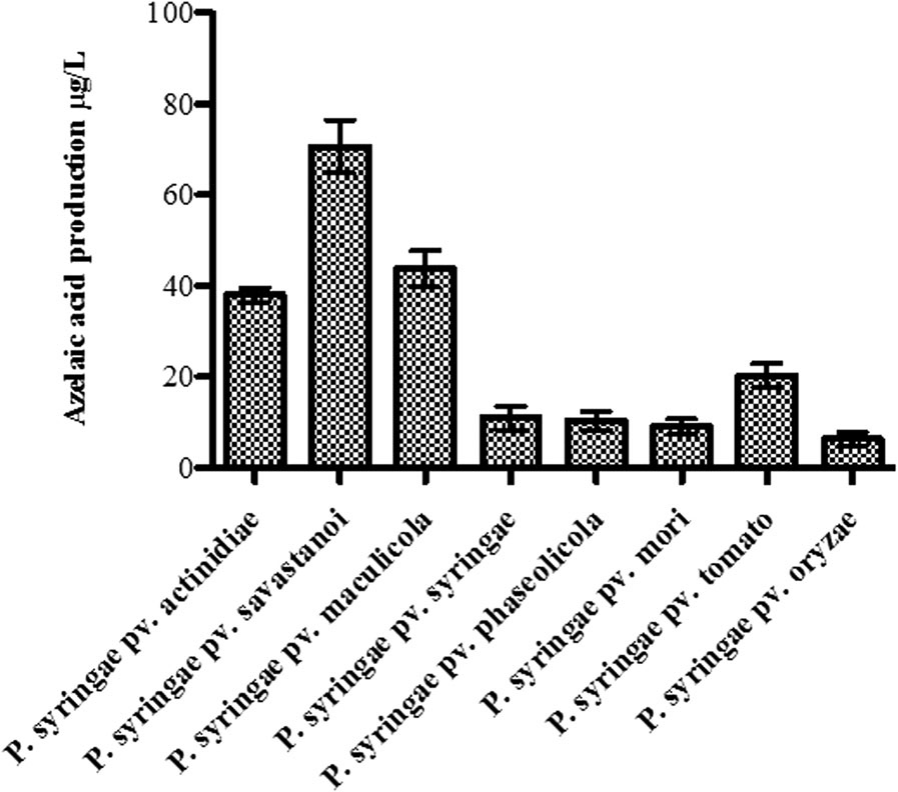
Production of azelaic acid from different *P. syringae* pathovars. Strains belonging to different *P. syringae* pathovars were cultured in M9 sucrose medium for 42 h and azelaic acid from spent medium was extracted and quantified. Bar indicates the mean and standard deviation of experiment performed in three biological replicates. Asterisks indicate significant difference between the azelaic acid production by PSA and different *P. syringae* pathovars (*P* < 0.05)

In order to investigate whether other members of the *P. syringae* pathovar group also contained azelaic acid in their spent supernatants, we tested 8 strains belonging to 8 different *P. syringae* pathovars. Importantly, all 8 strains appeared to be able to putatively produce azelaic acid and furthermore it was established that PSV produced 2-fold higher amounts of azelaic acid compared to the other strains tested (Fig. 5). On the contrary, we also tested for azelaic acid accumulation in a few other more distant species like *E. coli, B. subtilis* and *Rhizobium sp.*; and no traces of azelaic acid was detected in their spent supernatants (data not shown).

### Studies on azelaic acid acting as an intra-species signaling molecule

It was established from our previous studies that PSA does not produce AHL QS signaling molecules and to our knowledge, no signal involved in intraspecies signaling has been reported. Since azelaic acid was detected in the supernatants at concentration levels typical of QS signaling molecules (see above), it was of interest to determine whether azelaic acid could behave as a QS signal. We therefore performed a transcriptome analysis via an RNA seq experiment with the following rationale. Our hypothesis is that if azelaic acid behaves as a QS signal and is affecting gene expression in a cell-density dependent way, by providing it to an early logarithmic phase PSA culture this will probably result in a change of gene expression of target genes. This experiment was performed in LB medium since we determined that in PSA LB spent supernatants no azelaic could be detected (data not shown). RNAseq experiments were therefore performed in biological triplicates in the following two growth conditions. Firstly in PSA LB cultures supplemented at early logarithmic phase with 25 μM azelaic acid and secondly in PSA grown in LB supplemented at early logarithmic phase with only methanol since it was the solvent used to dissolve azelaic acid. After addition of these compounds in the two different set-ups, PSA was allowed to grow for 6 h and RNA was then purified at approximately mid-exponential phase. Comparing the RNAseq data with PSA supplemented with only methanol, the RNAseq data of PSA supplemented with azelaic acid resulted in the significant but rather low differential expression of 6 target loci (Additional file 6, namely: *gene 1818*, *gene3474*, *gene 3774*, *gene4120*, *gene4321*, *gene 4820*). As validation of the RNAseq data, the gene promoters of all the 6 loci were cloned in a promoter probe vector harboring a promoterless GFP reporter as described in the Methods section and gene promoter activity were determined in PSA with and without the exogenous addition of azelaic acid. With the exception of one locus (encoding a putative peptidase C1 protease) which responded to the presence of azelaic acid, all other gene promoters did not display altered expression under the different conditions tested (Additional file 7). It was concluded that azelaic acid did not show a role in intra-species signaling in this experimental set-up. It cannot be excluded that since in LB there is no production of azelaic acid by PSA, this growth condition could block or interfere with a putative signaling pathway.

## Discussion

Azelaic acid has been reported to have a biological role as a mobile signal in plants that induces systemic acquired resistance (SAR) upon *P. syringae* pathogen infection [30]. Upon infection by *P. syringae* on the model plant *A. thaliana*, the plant produces elevated levels of azelaic acid as detected in its vascular sap [30, 35]. Data presented in our study show that highest levels of in vitro production of azelaic acid occur in the presence of sucrose, this suggests that elevated levels of azelaic acid previously observed in *A. thaliana* may also be due to *P. syringae*. The sugars synthesized through photosynthesis are transported via the phloem and the total solute concentration is approximately 20% wt/wt consisting of approximately 80% sucrose [36]. We have however not presented any evidence showing that bacteria produce azelaic acid *in planta* as all our data is in vitro. Nevertheless, we can postulate that as sucrose is the most abundant carbon source available in the vascular sap, bacteria may utilize it to synthesize azelaic acid that could then have a role in plant defense responses. Therefore, it remains unclear how azelaic acid is synthesized *in planta* [37]. Lipid peroxidation (LPO), which is triggered by lipoxygenases (LOX) and reactive oxygen species (ROS), is a response to plant pathogen attack and has been proposed to be responsible for the presence of azelaic acid in plants. Lipidomics analysis of LPO in the plant-bacteria interaction of *A. thaliana* with *P. syringae* revealed a free radical catalyzed galactolipid fragmentation mechanism potentially responsible for the formation of azelaic acid and the biotin precursor pimelic acid [36]. Azelaic acid has therefore been proposed as a marker for LPO [37].

Our findings here raise the question on why *P. syringae* pathovars produces azelaic acid since it induces a defense response against the pathogen. Future work needs to clarify the possible biosynthesis of this molecule by *P. syringae in planta* and the potential role of azelaic acid in plant immunity in kiwifruit. *In planta* studies using azelaic acid production null mutants of *P. syringae* will be instrumental in understanding the role of azelaic acid in *P. syringae* and in plant-bacteria interaction. Furthermore, in this study we verified the extracellular presence of azelaic in *P. syringae* cultures and we did not test whether it is present intracellularly.

Our experiments unveiled that PSA and other *P. syringae* pathovars, were not utilizing azelaic acid as a carbon source (data not shown) and in addition do not indicate that it is a quorum sensing signal molecule. It cannot be excluded however that our experimental set-up used here in order to determine if azelaic acid could anticipate a quorum sensing response and activate gene expression could have its limitation. It must also be noted that we performed the RNAseq experiment in LB supplemented with azelaic acid in order to determine if the activation or repression of gene expression took place. It was established that PSA could not produce azelaic acid in LB media thus there is a possibility that the putative azelaic acid activated signal pathway might be blocked in LB medium. Secondly, anticipating the quorum by providing large quantities of signal at early phases of growth does not always lead to activation or repression of target genes [38, 39]. It cannot be unequivocally excluded therefore that azelaic acid is not a quorum sensing signal molecules and studies with a non-azelaic producing strain are necessary to support this conclusion. It is currently unknown whether PSA and other *P. syringae* pathovars possess a biosynthesis pathway for azelaic acid, if so, it is very laborious at this present time to isolate azelaic acid production mutants. It is well established that bacteria live as part of multispecies consortiums and most probably communicate with other species; a process called interspecies signaling [40, 41]. It is therefore also possible that azelaic acid could be an interspecies signal. Finally as mentioned above, it could also have a role as a defense molecule since it displays anti-microbial properties at certain concentrations [42, 43].

Industrially, azelaic acid is chemically synthesized and is used in the synthesis of many compounds such as polyamides, fragrances, adhesives, lubricants and polyesters [44–46]. The chemical synthesis of azelaic acid is currently performed in bulk by ozonolysis of oleic acid and this process has considerable drawbacks due to its non-eco-friendly nature [47–50]. Results presented here merit further investigations in order to explore possible bio-based production of azelaic acid by bacteria. It must be noted however that production levels by the wild type strains are extremely low; it cannot be excluded however that the production levels can considerably be increased via the identification and characterization of the putative biosynthesis pathway. Interestingly, a recent study also detected the presence of azelaic acid in the spent supernatants of lactic acid bacteria indicating that this feature could be common in bacteria [51].

## Conclusions

This work has shown the presence of azelaic acid in spent supernatants of *P. syringae* cultures and therefore calls for further investigations in order to determine its possible function as an bacterial or plant signal, as a secondary metabolite or as a byproduct of metabolism/transformation that ends up in the extracellular metabolome.

## Methods

### Bacterial strains and culture conditions

*P. syringae* pathovars used in this study are listed in Table 2 and were grown in Luria-Bertani (LB) broth or in M9 minimal medium (5.64 g Na_2_HPO_4_, 3 g KH_2_PO_4_, 0.5 g NaCl, 1 g NH_4_Cl, MgSO_4_ 2 mM and CaCl_2_ 0.1 mM) with 0.2% of the carbon source chosen among glucose, sucrose, fructose, maltose, xylose, or sorbitol [52]. The cultures were then incubated at room temperature with continuous shaking to reach saturation for up to 72 h. *E. coli* DH5α was grown in M9 medium with 0.2% glucose and 0.1 ml of a 0.5% vitamin B12 (thiamine) solution, 5 mL of a 20% casamino acids solution and incubated at 37 °C. *Bacillus subtilis* was grown in M9 medium comprising of 0.2% glucose with the addition of 10 mL of a trace element solution containing (per liter) 1.35 g FeCl_2_6H_2_O, 0.1 g MnCl_2_·H_2_O, 0.17 g ZnCl_2_, 0.043 g CuCl_2_·2H_2_O, 0.06 g CoCl_2_·6H_2_O, 0.06 g Na_2_MO_4_·2H_2_O [53]. *Rhizobium sullae* sp*.* nov HCNT1 was grown in a similar medium as of *E. coli* DH5α at 30 °C except that thiamine was replaced with biotin (1 mg/L) as indicated [54]. For cloning and promoter activity studies, *E. coli* DH5α and PSA were cultured using the antibiotics ampicillin 20 μg/mL and gentamycin 30 μg/mL.

**Table 2:** Bacterial strains used in this study

Name of the pathovars	Disease /Role in plants	Reference
*Pseudomonas syringae* pv*. actinidiae* (PSA 10.22)	Bacterial canker of kiwifruit (*Actinidia deliciosa* and *A. chinensis)*	[26]
*Pseudomonas syringae* pv*. savastanaoi* DAPP-PG 722	Olive knot disease in olive plant	[59]
*Pseudomonas syringae* pv. *maculicola* NCPPB 2039	Bacterial leaf spot on cruciferous plants	National collection of plant pathogenic bacteria
*Pseudomonas syringae* pv. *syringae B728a*	Causes bacterial brown spots on bean	[60]
*Pseudomonas syringae* pv. *phaseolicola* NCPPB 52	Causes halo blight of beans	National collection of plant pathogenic bacteria
*Pseudomonas syringae* pv. *mori* NCPPB 1034	Causes Bacterial Blight of Mulberries	National collection of plant pathogenic bacteria
*Pseudomonas syringae* pv. *tomato* NCPPB 4369	Causes bacterial speck of tomato.	National collection of plant pathogenic bacteria
*Pseudomonas syringae* pv. *oryzae* NCPPB 3683	Causes bacterial halo blight of rice	National collection of plant pathogenic bacteria
*Bacillus subtilis*	Non-pathogenic, but used widely as fungicide on vegetables and soya seeds	[61]
*E. coli* DH5α	F– *end*A1 *gln*V44 *thi*-1 *rec*A1 *rel*A1 *gyr*A96 *deo*R *nup*G *pur*B20 φ80d*lacZ*ΔM15 Δ(*lac*ZYA-*arg*F)U169, *hsd*R17(rK–mK+), λ– (For cloning purposes)	[62]
*Rhizobium sullae sp. nov* HCNT1	Root-nodule micro symbiont	[63]

### Extraction of the extracellular metabolome and azelaic acid from spent supernatants

The bacterial cells were pelleted by centrifugation at 10,816 x g for 10 min and the cell free supernatant was then collected and processed further as described [55]. Briefly, the cell free supernatant was acidified to pH 1 to 2 with conc. H_2_SO_4_ and extracted twice by adding an equal volume of diethyl ether (for the total extracellular metabolome, extraction was performed with ethyl acetate). The collected ether layers were combined, washed with distilled water (1 volume), and evaporated to dryness in rotary evaporation at 40 °C. A fraction of dried residue was dissolved in methanol (3 mL) and then subjected to HPLC and GC-MS analysis.

### GC-MS analysis

#### BF3 methanol derivatization

For total metabolome and azelaic acid analysis on GC-MS, a fraction of the dried residual samples were subjected to BF_3_ methanol derivatization prior to injection as follows: 2 mL of 10% BF_3_ methanol solution (Sigma, St Louis, MO, USA) was added to approximately 1 mg of the dried sample and incubated in a boiling water bath for 60 min. Subsequently, 2 mL of n-hexane was added and washed with milli-Q water (Millipore) at room temperature. The supernatant solvent was then separated by centrifugation.

#### GC-MS analysis of total extracellular metabolome

It was performed on Agilent technologies 6890 gas chromatography coupled to an Agilent technologies 5975B mass spectrometer. Compounds were eluted by a constant helium gas flow of 1.6 ml/min. With a split ratio of 4:1 and separated on a 60 m DB-WAX capillary column (0.25 film thickness, 0.25 mm internal diameter; Agilent Technologies, Santa Clara, USA). The temperature program was set at 100 °C for 2 min, followed by a ramp of 5 °C/min to the final temperature of 240 °C hold for 20 min. Mass spectra were acquired in the full scan mode collecting ions from 39 to 500 m/z using the electron impact ionization mode (e.i.) with electron energy of 70 eV. The acquired spectra were then compared with the spectra in the NIST-05 (NIST, Gaithersburg, USA) and in the WILEY 11 (Wiley, Hoboken, USA) libraries.

#### GC-MS analysis of HPLC purified fractions

It was carried out on Agilent technologies 7890A gas chromatograph coupled to an Agilent Technologies 5975C VL MSD equipped with an HP-1 capillary column (Agilent Technologies, 30 m). The temperature program used was as follows: at 70 °C for 2 min, 70–230 °C at 20 °C/ min. The MS data (total ion chromatogram TIC) were acquired in the full scan mode (m/z of 50–500) at a scan rate of 1000 amu using the electron impact ionization (e.i.) with an electron energy of 70 eV. The acquired spectrum was searched against the standard NIST-05 library/ WILEY 7 library.6 [56, 57].

### HPLC analysis

The crude extract (obtained as described above) was analyzed with a high performance liquid chromatography (HPLC) system (using a Varian 9050/9010 machine) equipped with an UV-visible detector. The separation was carried out using a 5 μm C18 reverse phase column (Agilent) and eluted with a gradient of water with 0.1% TFA as (A) and methanol with 0.1%TFA as (B) at a flow rate of 1.0 mL/min at 25 °C for 30 min. The gradient was created starting with 30% eluent B for 5 min, rising to 70% eluent B within 10 min, and returning to 30% eluent B within 5 min, followed by an equilibration of 10 min. The sample injected was 25 μL and the separated components were monitored by a UV detector at a range of 220 nm as described [56]. The data recording and processing was performed using a star chromatography workstation software. A standard curve was constructed ranging from 50 μg to 200 μg of azelaic acid. It was decided to use these concentrations since they were in the range of the concentrations present in the samples that were tested/prepared.

### Liquid chromatography - mass spectrometry (LC-MS)

Analyses were carried out using a Gilson 306 HPLC system equipped with an auto sampler. An Agilent Poroshell C18 (2.1 × 50 mm) column thermostatically controlled at 30 °C was used and the injected volume was 5 μL (3x full loop fill). The elution gradient was carried out with a binary solvent system consisting of 0.05% formic acid in H_2_O (solvent A) and MeCN (solvent B) at a constant flow-rate of 250 μL/min using a linear gradient profile from 0% B to 100% B in 30 min.

### Mass spectrometry

Electrospray mass spectrometry (ESI-MS) analysis was performed with an ion trap mass spectrometer (amazonSL, Bruker Daltonics). Samples were diluted 20 times in 10 mM ammonium acetate buffer pH 6.0 and 5 μL were analyzed by LC (see above). Mass spectra were acquired monitoring the 189/171 and 189/153 transitions in positive mode or the 187/125 transition in negative mode.

### NMR analysis

Azelaic acid standard and samples extracted from *P. syringae* pathovars were dissolved in 0.7 mL of CDCl_3_. Spectra were recorded on a 500 MHz VARIAN spectrometer operating at 25 °C, and subsequently processed using Mestre Nova software. Chemical shifts were referred to residual CHCl_3_ (at 7.26 ppm).

### Total RNA extraction, RNAseq experiment and analysis

Isolation of total RNA was carried out from three independent biological replicate cultures in two growth conditions of the wild type PSA strain; in one case a PSA culture was supplemented with methanol and in the other, PSA was supplemented with methanol containing azelaic acid. The methanol (20 μL) and methanol+azelaic acid (20 μL) were added to an equal number of PSA cells of (OD_600_ 0.04) in 25 mL LB medium. Growth parameters were set to 25 °C and 180 rpm of continuous shaking. After 2 h of incubation, which corresponds to early exponential phase, in one of the flasks a final concentration of 25 μM azelaic was added to the medium, and in other flask the same volume of methanol used to dissolve the azelaic acid was added as control. Thereafter cells were harvested from all the samples after 6 h of growth having similar cell number (OD_600_ 0.8) which corresponds to a mid-exponential growth phase. RNA was extracted and purified from the cells using the Ribopure bacteria RNA isolation kit (Ambion Inc., Austin, TX, U.S.A.) following the manufacturer’s instructions. Total RNA was treated with RNase-free DNase (Ambion, Life Technologies, and U.S.A.) and the purity of RNA was assessed by PCR on total RNA with GoTaq polymerase (Promega Corp.) using *PSA*R1 Fw and *PSA*R1 Rev. primers. rRNA depletion was performed and RNAseq was performed by IGA Technology Services Srl (Udine, Italy) where Illumina103 HiSeq2000 (Illumina Inc.) was used for sequencing. Resulting sequences were mapped against RefSeq assembly accession: AFTG00000000 [1] PSA CRAFRU 8.43 (cite PMID: 22535942 PMCID) using the BWA software (cite PMID: 20080505). Bioconductor libraries Genomic Features and DESeq2 (cite PMID 23950696 and 25,516,281) have been used to calculate gene expression levels and fold-changes between samples. The cutoff FDR-adjusted *P* value was 0.01, with a minimum two-fold change.

#### Accession number(s)

The original RNA-seq data have been submitted to the NCBI database under BioProject accession number PRJNA436767.

### Gene promoter studies

Transcriptional gene promoter activity studies of six promoters were performed in PSA. All constructs were made in the promoter probe plasmid vector pBBRGFPGm [58] which harbors the gene for gentamycin resistance and a promoterless *gfp* gene. The following gene promoters were used: *gene1818* encoding for a putative peptidase C1, *gene4120* encoding for a sugar ABC transporter, *gene 4321* coding for twitching motility protein, *gene4820* encoding for two component sensor histidine kinase protein, *gene3774* encoding for a histidine kinase, and *gene3474* encoding for a L-aspartate oxidase (Additional file 6). Promoter regions were amplified using primers listed in (Table 3) and were then transcriptionally fused to a promoterless *gfp* gene in vector pBBRGFP (Table 4). The gene promoter constructs pBBR-promoters-GFP were then electroporated into PSA WT as previously described [53]. Using the same growth conditions of the RNAseq experiment as described above, gene promoter activity was determined as a measurement of the intensity of GFP fluorescence at 510 nm on a Perkin Elmer EnVision 2104 micro plate reader.

**Table 3:** List of primers used in this study

Primer name	Sequence of primer	Amplified gene/template
prHKN220F	5’-AGGATCCTGGCAGTGCGCTGATAGCC-3′	DNA PSA wildtype
prHKN220R	5’-AGAATTCGCACAGCGCGCGCGATGAGC-3′	DNA PSA wildtype
prABC1220F	5’-AGGATCCGTGCAGGGCGCTTTCTTTG-3′	DNA PSA wildtype
prABC1220R	5’-AGAATTCCATTGGCGAGGCTTTGCTGTTC-3’	DNA PSA wild type
prPEP220F	5’-AGGATCCTTGCAGGTGGCGATTTGCGC-3’	DNA PSA wild type
prPEP220R	5’-AGAATTCAACCATGTGTGGAAGCGCCG-3’	DNA PSA wild type
prTWT220F	5’-AGGATCCAGCAGGCCTACGGACGC-3’	DNA PSA wild type
prTWT220R	5’-AGAATTTCCGACGCCCTGTTTGGC-3’	DNA PSA wild type
Pr2CSysFW	5’-AGGATCCGGATGGTGCCGGAAGC-3’	DNA PSA wild type
Pr2CSysRW	5’-AGAATTCTTTGCGGGAACCAGAGCGG-3’	DNA PSA wild type
prASP220F	5’-AGGATCCGCGCTGTACGCGCTCG-3’	DNA PSA wild type
prASP220R	5’-AGAATTTGCTCAGAACCGCAATCCGCA	DNA PSA wild type

**Table 4:** List of plasmids used in this study

Plasmids	Relevant characteristics	Reference
pBBRGFP-GM	pBBRMCS5 carrying promoterless *gfp* gen	[58]
pBBR-prom-3774-GFP	Promoter 3774 cloned in pBBRGFP	This study
pBBR-prom-4120-GFP	Promoter 4120 cloned in pBBRGFP	This study
pBBR-prom-1818-GFP	Promoter 1818 cloned in pBBRGFP	This study
pBBR-prom-4321-GFP	Promoter 4321 cloned in pBBRGFP	This study
pBBR-prom-4820-GFP	Promoter 4820 cloned in pBBRGFP	This study
pBBR-prom-3474-GFP	Promoter 3474 cloned in pBBRGFP	This study

### Statistical analysis

Statistical analysis was performed using the PRISM 5.0 software and that includes unpaired student’s t test. A *P* value of < 0.05 was considered significant.

## Additional files

Additional file 1: Table showing list of compounds identified by NIST library search of PSA metabolome cultured in M9 glucose medium. (DOCX 24 kb)

Additional file 2: Table showing list of compounds identified by NIST library search of PSV metabolome cultured in M9 glucose medium. (DOCX 21 kb)

Additional file 3: Table showing list of compounds identified by NIST library search of M9 glucose medium Control. (DOCX 25 kb)

Additional file 4: Figure showing Quantification of azelaic acid by LC-MS. LC-MS profile of standard azelaic acid and putative azelaic acid collected at RT 13 from HPLC (A). Quantification of isolated and purified azelaic acid by standard curve (B). In 5 μl of RT 13 sample injected, there is 0.476 μg of azelaic acid which can be translated to 19.992 μg/L. (PPTX 69 kb)

Additional file 5: Figure showing quantification of azelaic acid by HPLC on C18 reverse phase column. Quantification of azelaic acid (PSA produced) by standard curve A&B. (PPTX 51 kb)

Additional file 6: Table representing RNAseq expressed genes upon induction of azeliac acid. (DOCX 15 kb)

Additional file 7: Figure showing validation of the RNAseq-based expression by gene promoter studies. Promoters of selected gene were fused with promoter probe vector pBBRGFP-Gm and tested with similar concentration of azelaic acid which was used in RNAseq expression studies. Significant fold change from selected genes was observed only between pBGFPgene 1818/methanol and pBGFPgene 1818/azelaic acid. Data shown are the mean and standard deviation of experiment performed thrice in triplicate. (PPTX 99 kb)

## Abbreviations

^1^H-NMR: Proton nuclear magnetic resonance spectroscopy
COSY 2-D: Correlational two dimensional nuclear magnetic resonance spectroscopy
FDR: False discovery rate
GC-MS: Gas chromatography-mass spectrometry
GFP: Green fluorescent protein
HPLC: High performance liquid chromatography
NIST: National institute of standards and technology
NMR: Nuclear Magnetic resonance spectroscopy
PCR: Polymerase chain reaction
PSA: *Pseudomonas syringae* pv. *actinidiae*
PSV: *Pseudomonas savastanoi* pv. *savastanoi*
RefSeq: Reference sequence
RNAseq: RNA sequencing
BWA: Burrows-Wheeler Aligner
