# Supplementary material for: The spent culture supernatant of Pseudomonas syringae contains azelaic acid
Source: BMC Microbiol. 2018 Nov 28;18:199. doi: 10.1186/s12866-018-1352-z (PMC6264629; doi:10.1186/s12866-018-1352-z)
Supplement: Supplementary file 1 — Table showing list of compounds identified by NIST library search of PSA metabolome cultured in M9 glucose medium. (DOCX 24 kb) [file 12866_2018_1352_MOESM1_ESM.docx]

**Additional file 1**: **Table showing list of compounds identified by NIST library search of PSA metabolome cultured in M9 glucose medium**

| **S.no** | **R.T** | **Area%** | **Compound Name** |
| --- | --- | --- | --- |
| 1 | 7.33 | 0.14 | Cyclohexane, 1,1-dimethyl-2-propyl |
| 2 | 8.363 | 0.04 | Octanoic acid, methyl ester |
| 3 | 9.11 | 0.24 | 2-(3',3'-Dimethyl-1'-butyn-1'-yl)- 1-cyclohexenecarbaldehyde |
| 4 | 10.723 | 0.07 | 1-Octanol, 2-butyl |
| 5 | 11.03 | 0.14 | 2-Propanol, 1-[1-methyl-2-(2-prope nyloxy)ethoxy]- |
| 6 | 11.162 | 0.13 | Benzaldehyde |
| 7 | 11.357 | 0.1 | 6-Tridecene, 7-methyl- |
| 8 | 11.961 | 0.07 | methyl 3-acetylpropanoate |
| 9 | 12.371 | 0.04 | Butanedioic acid, dimethyl ester |
| 10 | 13.211 | 0.19 | Benzoic acid, methyl ester |
| 11 | 13.857 | 0.1 | 1-phenyl-ethanone |
| 12 | 14.335 | 0.1 | Docosane |
| 13 | 14.506 | 0.05 | Benzene, (1,2,2-trimethoxy-1-methy lethyl) |
| 14 | 15.744 | 0.11 | Cyclohexane, 1,2,4-trimethyl- |
| 15 | 15.884 | 0.19 | Benzoic acid, 3-methyl-, methyl ester |
| 16 | 16.041 | 0.17 | Benzeneacetic acid, methyl ester |
| 17 | 16.904 | 0.12 | Dodecanoic acid, methyl ester |
| 18 | 17.214 | 0.11 | Hexanedioic acid, dimethyl ester |
| 19 | 18.087 | 0.22 | Methyl ester of 2-hydroxyoctanoic acid |
| 20 | 20.232 | 0.14 | Cyclodecane |
| 21 | 20.688 | 0.05 | Methyl 8-methyl-decanoate |
| 22 | 20.747 | 0.06 | Phosphonofluoridic acid, (1-methyl ethyl)-, 2-methylpropyl ester |
| 23 | 20.901 | 0.46 | 3,5-Dioctoxyphenol |
| 24 | 21.147 | 0.4 | Methyl tetradecanoate |
| 25 | 21.536 | 0.08 | Octanedioic acid, dimethyl ester |
| 26 | 22.022 | 0.14 | Benzene, (3,3,3-trichloro-1-methylenepropyl) |
| 27 | 22.184 | 0.23 | Pentacosane |
| 28 | 22.368 | 0.5 | Methyl ester of 3-hydroxydecanoic acid |
| 29 | 22.555 | 0.15 | Phenol, 3-methyl- |
| 30 | 23.211 | 0.58 | Cyclopentane, 1,2,3-trimethyl-, (1 .alpha.,2.alpha.,3.beta.)- |
| 31 | 23.588 | 0.26 | Azelaic acid, dimethyl ester |
| 32 | 23.703 | 0.05 | 1-Dichloromethyl(dimethyl)silyloxy methyl-4-methoxybenzene |
| 33 | 24.2 | 0.15 | Acetic acid, trifluoro-, dodecyl ester |
| 34 | 24.676 | 0.34 | 1-Propene-1,2,3-tricarboxylic acid, trimethyl ester, € |
| 35 | 24.828 | 0.08 | Decane, 2-methyl- $$ 2-methyl-decane |
| 36 | 25.215 | 16.31 | Hexadecanoic acid, methyl ester |
| 37 | 25.692 | 12.67 | 9-Hexadecenoic acid, methyl ester |
| 38 | 25.885 | 0.24 | Benzene, 1,1'-(1,3-propanediyl)bis- $$ (3-PHENYLPROPYL)BENZENE |
| 39 | 26.049 | 1.08 | Benzenepropanoic acid, .alpha.-hydroxy-, methyl ester |
| 40 | 26.321 | 0.19 | Butyl 2,5,8,11,14,17,20,23,26-nonaoxaoctacosan-28-oate |
| 41 | 26.504 | 0.53 | Phenol, 2,4-bis(1,1-dimethylethyl) |
| 42 | 26.609 | 0.25 | 1,2-benzene-dicarboxylic acid, dim ethyl ester |
| 43 | 26.762 | 0.21 | 1-Pentadecanol acetate |
| 44 | 26.934 | 0.34 | Benzene, 1,1'-(1,2-cyclobutanediyl)bis-, trans- |
| 45 | 27.045 | 0.13 | 21-KRONE-7 |
| 46 | 27.404 | 0.35 | Valeraldehyde, (o-nitrophenyl)hydrazone |
| 47 | 27.559 | 0.12 | 1,4,7,10,13,16-Hexaoxacyclooctadecane |
| 48 | 27.856 | 0.52 | 2,4-Diphenyl-1-butene $$ (1-methylene-3-phenyl-propyl)benzene |
| 49 | 28.122 | 0.17 | 1,4,7,10,13,16-Hexaoxacyclooctadecane |
| 50 | 28.245 | 0.21 | 21-KRONE-7 $$ 1,4,7,10,13,16,19-heptaoxacycloheneicosane |
| 51 | 28.407 | 2.55 | [18O]-TRIMETHYL 2-HYDROXY-1,2,3-PR OPANETRICARBOXYLATE |
| 52 | 28.522 | 0.69 | Benzene, 1,1'-(1,2-cyclobutanediyl )bis-, trans- |
| 53 | 28.775 | 6.33 | Octadecanoic acid, methyl ester |
| 54 | 28.972 | 0.35 | Naphthalene, 1,2,3,4-tetrahydro-1-phenyl- |
| 55 | 29.276 | 11.07 | 9-Octadecenoic acid (Z)-, methylester |
| 56 | 29.605 | 0.5 | 1,2-Diphenylcyclopropane |
| 57 | 29.75 | 0.33 | 2-[2-[2-[2-[2-[2-[2-[2-[2-[2-(2-Hy droxyethoxy)ethoxy]ethoxy]ethoxy]e thoxy]ethoxy]ethoxy]ethoxy]ethoxy]  ethoxy]ethanol |
| 58 | 29.965 | 0.72 | 2,5-Cyclohexadien-1-one, 2,6-di-tert-butyl-4-(hydroxymethylene)- |
| 59 | 30.099 | 1.25 | Benzene, 1,1'-(3-methyl-1-propene- 1,3-diyl)bis- |
| 60 | 30.228 | 0.34 | 1,2-Benzenedicarboxylic acid, butyl methyl ester |
| 61 | 30.701 | 0.48 | 1,2-Benzenedicarboxylic acid, dihester |
| 62 | 30.828 | 0.37 | Heptacosane |
| 63 | 31.053 | 1.47 | Naphthalene, 1,2-dihydro-1-phenyl- |
| 64 | 31.306 | 1.68 | METHYL-3-(3,5-DITERTBUTYL-4-HYDROX YPHENYL) PROPIONATE |
| 65 | 31.5 | 0.46 | Nonadecane |
| 66 | 31.63 | 0.46 | 2-Hexadecanol |
| 67 | 31.721 | 0.36 | Docosane |
| 68 | 31.908 | 0.7 | 2-Hexadecanol |
| 69 | 32.271 | 0.82 | 21-KRONE-7 |
| 70 | 32.752 | 0.77 | Heptacosane |
| 71 | 32.998 | 2.46 | Octadecanoic acid, 11-methoxy-, methyl ester |
| 72 | 33.381 | 1.96 | Glutaric acid, 2-hexyl octyl ester |
| 73 | 33.656 | 0.59 | Dibutyl phthalate |
| 74 | 34.06 | 1.54 | 21-KRONE-7 |
| 75 | 34.659 | 1.25 | 2,5-dimethoxybenzene-1,4-diol |
| 76 | 35.473 | 0.47 | 1H-Pyrrolo[2,3-d]pyrimidin-4-amine |
| 77 | 35.575 | 0.31 | 21-KRONE-7 |
| 78 | 35.914 | 0.27 | .beta.-Phenylpropiophenone |
| 79 | 38.231 | 2.68 | Methyl 2-(2-oxo-4-quinolyl)formate |
| 80 | 38.556 | 1.82 | Hexadecanoic acid |
| 81 | 39.97 | 1.78 | Benzoic acid, 4-hydroxy-, methyl ester |
| 82 | 41.6 | 0.6 | 1H-Pyrazole-4-carbonitrile, 1-phenyl-5- |
| 83 | 50.104 | 1.51 | Anisole, p-(phenylethynyl)- |
