# Supplementary material for: The spent culture supernatant of Pseudomonas syringae contains azelaic acid
Source: BMC Microbiol. 2018 Nov 28;18:199. doi: 10.1186/s12866-018-1352-z (PMC6264629; doi:10.1186/s12866-018-1352-z)
Supplement: Supplementary file 2 — Table showing list of compounds identified by NIST library search of PSV metabolome cultured in M9 glucose medium. (DOCX 21 kb) [file 12866_2018_1352_MOESM2_ESM.docx]

**Additional file 2**: **Table** **showing list of compounds identified by NIST library search of PSV metabolome cultured in M9 glucose medium**

| \| S.No \| R.T \| Area \| Compound Name \| \| --- \| --- \| --- \| --- \| \| 1 \| 7.134 \| 0.34 \| Pyridine, 3-methyl- $$ 3-Picoline \| \| 2 \| 9.302 \| 0.16 \| Acetic acid $$ ACETASOL $$ ACETATE \| \| 3 \| 10.183 \| 0.43 \| 2-Propenoic acid, 2-ethylhexyl est \| \| 4 \| 13.9 \| 0.42 \| Benzaldehyde, 4-methyl- $$ 4-Methy 27451 000104-87-0 97 \| \| 5 \| 16.046 \| 0.37 \| Benzeneacetic acid, methyl ester \| \| 6 \| 16.9 \| 0.18 \| Dodecanoic acid, methyl ester \| \| 7 \| 18.092 \| 0.14 \| Methyl 3-hydroxytetradecanoate \| \| 8 \| 20.229 \| 0.52 \| Cyclodecane \| \| 9 \| 20.9 \| 0.22 \| Phenol $$ 3,5-Dioctoxyphenol \| \| 10 \| 21.148 \| 0.61 \| 12-methyl-tridecanoic acid methyl ester \| \| 11 \| 22.381 \| 5.29 \| Methyl 3-hydroxytetradecanoate \| \| 12 \| 23.214 \| 1.42 \| Cyclopentane, 1, 2,3-trimethyl-, (1 .alpha.,2.alpha.,3.beta.) \| \| 13 \| 23.5 \| 0.33 \| Azelaic acid, dimethyl ester \| \| 14 \| 24.196 \| 0.38 \| Trichloroacetic acid, hexadecyl es ter \| \| 15 \| 24.675 \| 0.47 \| Glutaric acid, ethyl 2-methylpentyester \| \| 16 \| 25.113 \| 11.4 \| Hexadecanoic acid, methyl ester \| \| 17 \| 25.537 \| 1.46 \| Benzoic acid, 2-amino-, methyl ester \| \| 18 \| 25.706 \| 0.25 \| Nonadecane $$ n-Nonadecane \| \| 19 \| 26.084 \| 0.2 \| 2(1H)-Pyridinone-5,6-d2, 4-methoxy-1-methyl \| \| 20 \| 26.317 \| 0.35 \| Octadecanoic acid, 3-hydroxy-, met hyl ester \| \| 21 \| 26.507 \| 0.2 \| Phenol, 2,4-bis(1,1-dimethylethyl) \| \| 22 \| 26.6 \| 0.57 \| Dimethyl phthalate $$ 1,2-Benzenedicarboxylic acid, dimethyl ester \| \| 23 \| 27.858 \| 0.3 \| 1-Nonadecene \| \| 24 \| 28.728 \| 4.82 \| Octadecanoic acid, methyl ester \| \| 25 \| 28.875 \| 0.22 \| 21-KRONE-7 $$ 1,4,7,10,13,16,19-he ptaoxacycloheneicosane \| \| 26 \| 29.356 \| 7.58 \| 2-Methoxy-6,7,8,9-tetrahydro-9-methylpyrido[1,2-a]indole \| \| 27 \| 29.747 \| 0.75 \| Octaethylene glycol monododecyl et her \| \| 28 \| 30.147 \| 0.75 \| Octaethylene glycol monododecyl ether \| \| 29 \| 31.08 \| 2.5 \| 4-(4-BROMO-2-METHYLANILINO)-4-OXOB UTANOIC ACID 93 \| \| 30 \| 31.3 \| 2.16 \| 1-Eicosene \| \| 31 \| 31.9 \| 2.86 \| 1H-Azepine-1-carboxaldehyde, hexah ydro-2-(methylphenyl) \| \| 32 \| 32.28 \| 1.86 \| 1,2-Propanediol, 3-(butylthio)- 21-KRONE-7 \| \| 33 \| 32.775 \| 0.95 \| 21-KRONE-7 $$ 1,4,7,10,13,16,19-he ptaoxacycloheneicosane \| \| 34 \| 32.971 \| 5.76 \| 4-(2-Ethoxyvinyl)-2-methylisoquino lin-1(2H)-one \| \| 35 \| 33.294 \| 0.5 \| 21-KRONE-7 $$ 1,4,7,10,13,16,19-heptaoxacycloheneicosane \| \| 36 \| 33.386 \| 2.37 \| Glutaric acid, isobutyl tridec-2-ynyl ester \| \| 37 \| 33.9 \| 4.3 \| Methyl ester of 3-hydroxydecanoic acid \| \| 38 \| 34.039 \| 1.35 \| 21-KRONE-7 $$ 1,4,7,10,13,16,19-heptaoxacycloheneicosane \| \| 39 \| 34.259 \| 0.49 \| Octaethylene glycol monododecyl ether \| \| 40 \| 34.4 \| 0.92 \| Thiazole, 5-ethyl-2-methyl- $$ 5-Ethyl-2-methyl-1,3-thiazole \| \| 41 \| 35.484 \| 2.11 \| 2-(5-acetyl-2-furyl)-1,4-naphthoquinone \| \| 42 \| 36.363 \| 0.44 \| 21-KRONE-7 $$ 1,4,7,10,13,16,19-heptaoxacycloheneicosane \| \| 43 \| 36.478 \| 0.65 \| Tetraethylene glycol monododecylether \| \| 44 \| 37.015 \| 0.5 \| 2-[2-[2-[2-[2-[2-[2-(2-Hydroxyethoxy)ethoxy]ethoxy]ethoxy]ethoxy]eth oxy]ethoxy]ethanol $$ Octaethyleneglycol \| \| 45 \| 38.425 \| 0.23 \| ,4,7,10,13,16-Hexaoxacyclooctadecane \| \| 46 \| 38.571 \| 6.25 \| Hexadecanoic acid $$ 1-Pentadecanecarboxylic acid \| \| 47 \| 39.459 \| 0.91 \| 2-[2-[2-[2-[2-[2-[2-(2-Methoxyethoxy)ethoxy]ethoxy]ethoxy]ethoxy]eth oxy]ethoxy]ethanol \| \| 48 \| 40.653 \| 0.86 \| 1,4,7,10,13,16-Hexaoxacyclooctadecane \| \| 49 \| 43.34 \| 0.71 \| ,4,7,10,13,16-Hexaoxanonadecane18-propyl- \| \| 50 \| 46.855 \| 4.45 \| Octadecanoic acid \| \| 51 \| 47.508 \| 0.56 \| 1,4,7,10,13,16-Hexaoxacyclooctadecane \| \| 52 \| 48.748 \| 0.39 \| 1,4,7,10,13,16-Hexaoxacyclooctadecane \| \| 53 \| 50.056 \| 1.72 \| 6-Chloro-N-cyano-N-methoxymethyl-N ',N'-dimethyl-1,3,5-triazine-2,4-diamine \| |  |  |  |  |
| --- | --- | --- | --- | --- | --- | --- | --- | --- | --- | --- | --- | --- | --- | --- | --- | --- | --- | --- | --- | --- | --- | --- | --- | --- | --- | --- | --- | --- | --- | --- | --- | --- | --- | --- | --- | --- | --- | --- | --- | --- | --- | --- | --- | --- | --- | --- | --- | --- | --- | --- | --- | --- | --- | --- | --- | --- | --- | --- | --- | --- | --- | --- | --- | --- | --- | --- | --- | --- | --- | --- | --- | --- | --- | --- | --- | --- | --- | --- | --- | --- | --- | --- | --- | --- | --- | --- | --- | --- | --- | --- | --- | --- | --- | --- | --- | --- | --- | --- | --- | --- | --- | --- | --- | --- | --- | --- | --- | --- | --- | --- | --- | --- | --- | --- | --- | --- | --- | --- | --- | --- | --- | --- | --- | --- | --- | --- | --- | --- | --- | --- | --- | --- | --- | --- | --- | --- | --- | --- | --- | --- | --- | --- | --- | --- | --- | --- | --- | --- | --- | --- | --- | --- | --- | --- | --- | --- | --- | --- | --- | --- | --- | --- | --- | --- | --- | --- | --- | --- | --- | --- | --- | --- | --- | --- | --- | --- | --- | --- | --- | --- | --- | --- | --- | --- | --- | --- | --- | --- | --- | --- | --- | --- | --- | --- | --- | --- | --- | --- | --- | --- | --- | --- | --- | --- | --- | --- | --- | --- | --- | --- | --- | --- | --- | --- | --- | --- | --- | --- | --- | --- |
|  |  |  |  |  |
