# Supplementary material for: The spent culture supernatant of Pseudomonas syringae contains azelaic acid
Source: BMC Microbiol. 2018 Nov 28;18:199. doi: 10.1186/s12866-018-1352-z (PMC6264629; doi:10.1186/s12866-018-1352-z)
Supplement: Supplementary file 3 — Table showing list of compounds identified by NIST library search of M9 glucose medium Control. (DOCX 25 kb) [file 12866_2018_1352_MOESM3_ESM.docx]

|  | |  |  |  | |  | |
| --- | --- | --- | --- | --- | --- | --- | --- |
| **Additional file 3**: **Table** **showing list of compounds identified by NIST library search of M9 glucose medium (Control)** | |  |  |  | |  | |
| \| S.no \| RT \| Area % \| Compound name \| \| --- \| --- \| --- \| --- \| \| 1 \| 5.203 \| 0.08 \| Methanesulphonic anhydride \| \| 2 \| 5.676 \| 0.04 \| Dodecane, 4,6-dimethyl \| \| 3 \| 5.749 \| 0.05 \| TETRADECANE \| \| 4 \| 5.928 \| 0.15 \| Tridecanol, 2-ethyl-2-methyl \| \| 5 \| 6.685 \| 0.11 \| 5-ETHYL-2-METHYLOCTANE \| \| 6 \| 7.049 \| 0.22 \| 2-Decene, 7-methyl-, (Z)- \| \| 7 \| 7.188 \| 0.27 \| Cyclopentanone, 2-acetyl \| \| 8 \| 7.331 \| 0.2 \| Cyclopentanone, 2-acetyl \| \| 9 \| 8.388 \| 4.72 \| Octanoic acid, methyl ester \| \| 10 \| 8.737 \| 0.1 \| Decane, 2-methyl \| \| 11 \| 8.925 \| 0.16 \| Hexadecane, 4-methyl \| \| 12 \| 9.115 \| 0.34 \| Benzene, 1,3-bis \| \| 13 \| 9.286 \| 0.2 \| 5S,9S)-5,9-dimethylpentadecane \| \| 14 \| 10.045 \| 0.07 \| DIPROPYLENEGLYCOL METHYL ETHER \| \| 15 \| 10.196 \| 0.29 \| UNDECANE, 5-METHYL \| \| 16 \| 10.722 \| 0.14 \| Triacontane \| \| 17 \| 10.936 \| 0.16 \| 4,4-heptafluorobutanoate \| \| 18 \| 11.034 \| 0.47 \| Dipropylene glycol monomethyl ether \| \| 19 \| 11.362 \| 0.21 \| 6-Tridecene, 7-methyl-6tridecene 62 \| \| 20 \| 11.585 \| 0.13 \| 1-Hexene, 3,3-dimethyl- \| \| 21 \| 12.308 \| 0.03 \| Phosphoric acid, trimethyl ester \| \| 22 \| 13.013 \| 0.13 \| Decane, 2-methyl- $$ 2-methyl-decane \| \| 23 \| 13.194 \| 0.39 \| Docosane \| \| 24 \| 13.831 \| 0.53 \| o-Methylbenzaldehyde \| \| 25 \| 14.301 \| 0.27 \| ICOSANE \| \| 26 \| 14.857 \| 0.25 \| n-Icosane \| \| 27 \| 15.106 \| 0.17 \| (2Z)-4,5-DIMETHYL-2-UNDECENE \| \| 28 \| 15.305 \| 0.3 \| Nonadecyl trifluoroacetate \| \| 29 \| 15.575 \| 0.44 \| 5-dimethylundec-2-ene \| \| 30 \| 15.711 \| 0.33 \| Triallylsilane \| \| 31 \| 16.066 \| 0.17 \| Cyclohexane, 1,2,4-trimethyl \| \| 32 \| 16.322 \| 0.16 \| 1,2,4-trimethylcyclohexane \| \| 33 \| 16.901 \| 0.05 \| Eicosane \| \| 34 \| 17.21 \| 0.05 \| Eicosane \| \| 35 \| 17.374 \| 0.14 \| Pentadecane, 3-methyl \| \| 36 \| 17.562 \| 0.05 \| Hexacosane \| \| 37 \| 18.358 \| 0.16 \| Eicosane \| \| 38 \| 19.27 \| 0.07 \| 2-methylhexacosane \| \| 39 \| 19.52 \| 0.17 \| Nonadecane, 9-methyl- \| \| 40 \| 19.797 \| 0.18 \| Cyclohexane, 1,2,4-trimethyl \| \| 41 \| 20.3 \| 0.17 \| 4,4-Dichloro-2-phenyl-1-butene \| \| 42 \| 20.896 \| 0.42 \| Phenol \| \| 43 \| 21.142 \| 0.16 \| Methyl isomyristate \| \| 44 \| 21.536 \| 0.04 \| 2-Butenoic acid, 2-methoxy-3-methy \| \| 45 \| 21.898 \| 0.23 \| Octanoic acid \| \| 46 \| 22.118 \| 0.19 \| Heptacosane \| \| 47 \| 22.544 \| 0.05 \| Tetratriacontane \| \| 48 \| 23.001 \| 0.11 \| Eicosane \| \| 49 \| 23.208 \| 0.55 \| 1-Butoxy-2-ethylhexane \| \| 50 \| 23.446 \| 0.18 \| 1-Dodecanol, 2-octyl- \| \| 51 \| 23.585 \| 0.21 \| 5-Methyl-Z-5-docosene \| \| 52 \| 23.809 \| 0.11 \| Decane, 2-methyl \| \| 53 \| 23.928 \| 0.16 \| 1-ETHYL-2-PROPYLCYCLOHEXANE \| \| 54 \| 24.248 \| 0.37 \| Cyclohexane, 1-ethyl-2-propyl- \| \| 55 \| 24.427 \| 0.1 \| 8,9-Dimethoxy-1,2,3,4-ttrahydronap \| \| 56 \| 24.665 \| 0.26 \| Lepidine \| \| 57 \| 24.828 \| 0.55 \| Hydroxylamine, O-(1-naphthalenylme 126494 054484-68-3 22 \| \| 58 \| 25.092 \| 1.61 \| Hexadecanoic acid, methyl ester \| \| 59 \| 25.391 \| 0.15 \| Decane, 2-methyl- \| \| 60 \| 25.554 \| 0.31 \| Decane, 2-methyl \| \| 61 \| 26.499 \| 0.2 \| 1-HYDROXY-2,4-DI-TERT-BUTYLBE \| \| 62 \| 26.598 \| 0.14 \| Phthalic acid, dimethyl ester \| \| 63 \| 26.944 \| 0.12 \| Naphthalene, 2,7-dimethyl \| \| 64 \| 27.036 \| 0.12 \| 2,6-Dimethyl-2-decen-8-ol \| \| 65 \| 27.575 \| 0.1 \| Ethylene oxide cyclic hexamer \| \| 66 \| 27.842 \| 0.27 \| Ethylene oxide cyclic hexamer \| \| 67 \| 28.715 \| 4.83 \| Methyl stearate \| \| 68 \| 28.863 \| 0.81 \| 4-Methoxy-2-pyridinethiol 1-oxide \| \| 69 \| 28.957 \| 0.72 \| 2-METHYLAMINOTHIOXO-THIOphene \| \| 70 \| 29.097 \| 1.65 \| 4-Methoxy-2-pyridinethiol 1-oxide \| \| 71 \| 29.192 \| 0.83 \| 4-Methoxy-2-pyridinethiol 1-oxide \| \| 72 \| 29.282 \| 6.64 \| 2,4,5-Trifluorobenzonitrile \| \| 73 \| 30.391 \| 1.11 \| Benzoic acid, 4-methyl \| \| 74 \| 30.692 \| 0.29 \| Heptacosane \| \| 75 \| 31.309 \| 3.04 \| Docosane \| \| 76 \| 31.726 \| 2 \| Eicosane \| \| 77 \| 32.76 \| 0.78 \| n-eicosanen-icosane \| \| 78 \| 32.905 \| 0.61 \| Heneicosane \| \| 79 \| 33.134 \| 0.35 \| Octacosane \| \| 80 \| 33.278 \| 0.23 \| 21-KRONE-7 \| \| 81 \| 33.359 \| 0.49 \| 2H-Pyran-2,5(6H)-dione, 3-methoxy- 535252 131146-48-0 90 \| \| 82 \| 33.87 \| 0.27 \| Ethylene oxide cyclic hexamer \| \| 83 \| 34.071 \| 1.37 \| Heneicosane \| \| 84 \| 34.492 \| 0.68 \| -ETHYL-2-METHYL-1,3-THIAZOLE \| \| 85 \| 35.235 \| 0.48 \| 1,2-Propanediol, 3-(butylthio)- \| \| 86 \| 35.489 \| 5.35 \| Methyl-d3 1,2-Dimethyl-2-propenyl 11601 2000011-60-ether \| \| 87 \| 36.164 \| 0.04 \| Ethylene oxide cyclic hexamer \| \| 88 \| 37 \| 0.1 \| 1-methyl-4-(2,4,5-trifluorophenyl)1H-pyrazole-3-carbonitrile \| \| 89 \| 37.937 \| 0.09 \| 2-AMINO-7-PHENYL-18-NAPHTHYRIDIN-5-ONE $, \| \| 90 \| 38.581 \| 3.74 \| Hexadecanoic acid \| \| 91 \| 39.557 \| 0.12 \| 21-KRONE-7 \| |  |  |  | |  | |  |
|  |  |  |  | |  | |  |
|  |  |  |  | |  | |  |
