# Supplementary material for: The spent culture supernatant of Pseudomonas syringae contains azelaic acid
Source: BMC Microbiol. 2018 Nov 28;18:199. doi: 10.1186/s12866-018-1352-z (PMC6264629; doi:10.1186/s12866-018-1352-z)
Supplement: Supplementary file 4 — Figure showing Quantification of azelaic acid by LC-MS. LC-MS profile of standard azelaic acid and putative azelaic acid collected at RT 13 from HPLC (A). Quantification of isolated and purified azelaic acid by standard curve (B). In 5 μl of RT 13 sample injected, there is 0.476 μg of azelaic acid which can be translated to 19.992 μg/L. (PPTX 69 kb) [file 12866_2018_1352_MOESM4_ESM.pptx]

## Slide 1
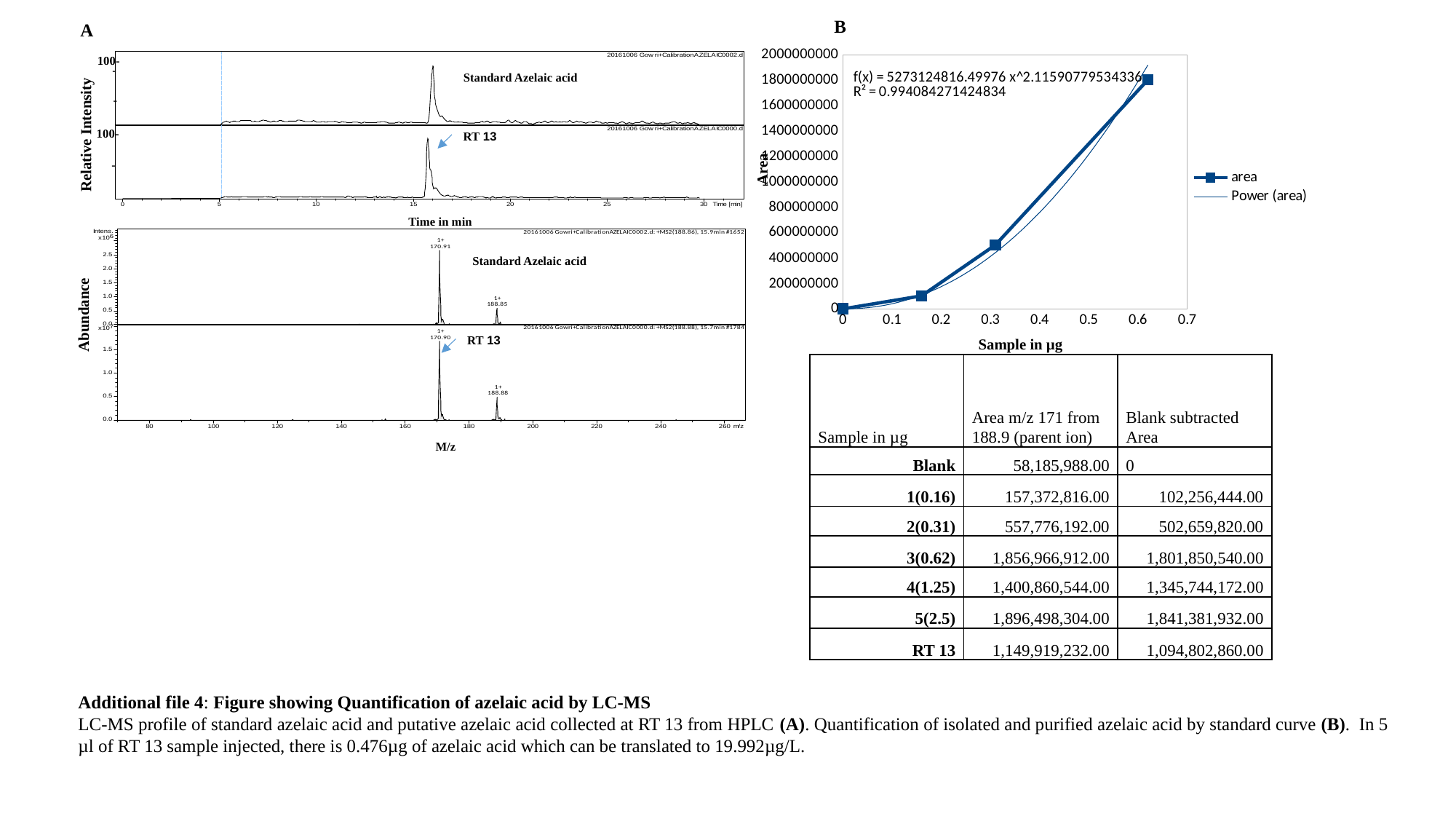

B
A
100-
-
Standard Azelaic acid
-
Relative Intensity
100-
RT 13
-
Time in min
Standard Azelaic acid
RT 13
M/z
### Chart
| Category | area |
|---|---|Area
Abundance
Sample in µg
| Sample in µg | Area m/z 171 from 188.9 (parent ion) | Blank subtracted Area |
| --- | --- | --- |
| Blank | 58,185,988.00 | 0 |
| 1(0.16) | 157,372,816.00 | 102,256,444.00 |
| 2(0.31) | 557,776,192.00 | 502,659,820.00 |
| 3(0.62) | 1,856,966,912.00 | 1,801,850,540.00 |
| 4(1.25) | 1,400,860,544.00 | 1,345,744,172.00 |
| 5(2.5) | 1,896,498,304.00 | 1,841,381,932.00 |
| RT 13 | 1,149,919,232.00 | 1,094,802,860.00 |
Additional file 4: Figure showing Quantification of azelaic acid by LC-MS
LC-MS profile of standard azelaic acid and putative azelaic acid collected at RT 13 from HPLC (A). Quantification of isolated and purified azelaic acid by standard curve (B). In 5 µl of RT 13 sample injected, there is 0.476µg of azelaic acid which can be translated to 19.992µg/L.
