# Supplementary material for: The spent culture supernatant of Pseudomonas syringae contains azelaic acid
Source: BMC Microbiol. 2018 Nov 28;18:199. doi: 10.1186/s12866-018-1352-z (PMC6264629; doi:10.1186/s12866-018-1352-z)
Supplement: Supplementary file 5 — Figure showing quantification of azelaic acid by HPLC on C18 reverse phase column. Quantification of azelaic acid (PSA produced) by standard curve A&B. (PPTX 51 kb) [file 12866_2018_1352_MOESM5_ESM.pptx]

## Slide 1
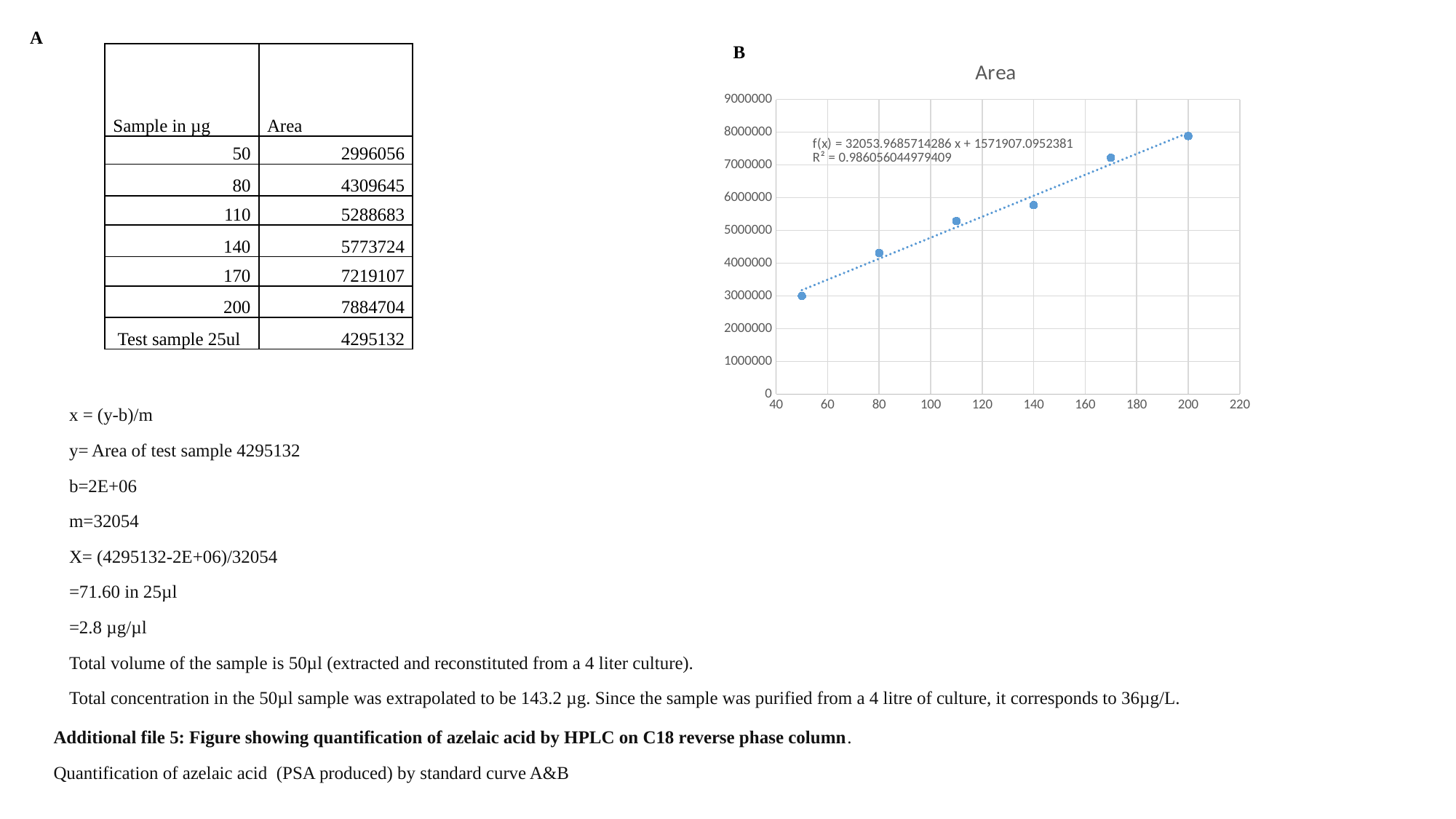

A
B
### Chart:
| Category | Area |
|---|---|| Sample in µg | Area |
| --- | --- |
| 50 | 2996056 |
| 80 | 4309645 |
| 110 | 5288683 |
| 140 | 5773724 |
| 170 | 7219107 |
| 200 | 7884704 |
| Test sample 25ul | 4295132 |
x = (y-b)/m
y= Area of test sample 4295132
b=2E+06
m=32054
X= (4295132-2E+06)/32054
=71.60 in 25µl
=2.8 µg/µl
Total volume of the sample is 50µl (extracted and reconstituted from a 4 liter culture).
Total concentration in the 50µl sample was extrapolated to be 143.2 µg. Since the sample was purified from a 4 litre of culture, it corresponds to 36µg/L.
Additional file 5: Figure showing quantification of azelaic acid by HPLC on C18 reverse phase column.
Quantification of azelaic acid (PSA produced) by standard curve A&B
