# Supplementary material for: The spent culture supernatant of Pseudomonas syringae contains azelaic acid
Source: BMC Microbiol. 2018 Nov 28;18:199. doi: 10.1186/s12866-018-1352-z (PMC6264629; doi:10.1186/s12866-018-1352-z)
Supplement: Supplementary file 6 — Table representing RNAseq expressed genes upon induction of azeliac acid. (DOCX 15 kb) [file 12866_2018_1352_MOESM6_ESM.docx]

|  |  |  |  |  |
| --- | --- | --- | --- | --- |
| **Additional file 6: Table representing RNAseq expressed genes upon induction of azeliac acid** |  |  |  |  |

| S.no | Gene Accession | Fold change Azelaic acid/Methanol | Protein | Function/Role |
| --- | --- | --- | --- | --- |
| 1 | gene3774 | 1.53685 | Histidine kinase | Signal transduction/ phosphorylation |
| 2 | gene4120 | 1.70449 | Sugar ABC transporter | Membrane/Transport |
| 3 | gene1818 | 1.73212 | Multi species peptidase C1 | Cysteine-type peptidase activity |
| 4 | gene4321 | 1.99898 | Twitching motility protein | Transport/ATP binding |
| 5 | gene4820 | 2.0652 | Two component sensor histidine kinase protein | Signal transduction/ phosphorylation |
| 6 | gene3474 | 2.78624 | L-aspartate oxidase | NAD biosynthetic process/ oxidation-reduction process |
