# Supplementary material for: The spent culture supernatant of Pseudomonas syringae contains azelaic acid
Source: BMC Microbiol. 2018 Nov 28;18:199. doi: 10.1186/s12866-018-1352-z (PMC6264629; doi:10.1186/s12866-018-1352-z)
Supplement: Supplementary file 7 — Figure showing validation of the RNAseq-based expression by gene promoter studies. Promoters of selected gene were fused with promoter probe vector pBBRGFP-Gm and tested with similar concentration of azelaic acid which was used in RNAseq expression studies. Significant fold change from selected genes was observed only between pBGFPgene 1818/methanol and pBGFPgene 1818/azelaic acid. Data shown are the mean and standard deviation of experiment performed thrice in triplicate. (PPTX 99 kb) [file 12866_2018_1352_MOESM7_ESM.pptx]

## Slide 1
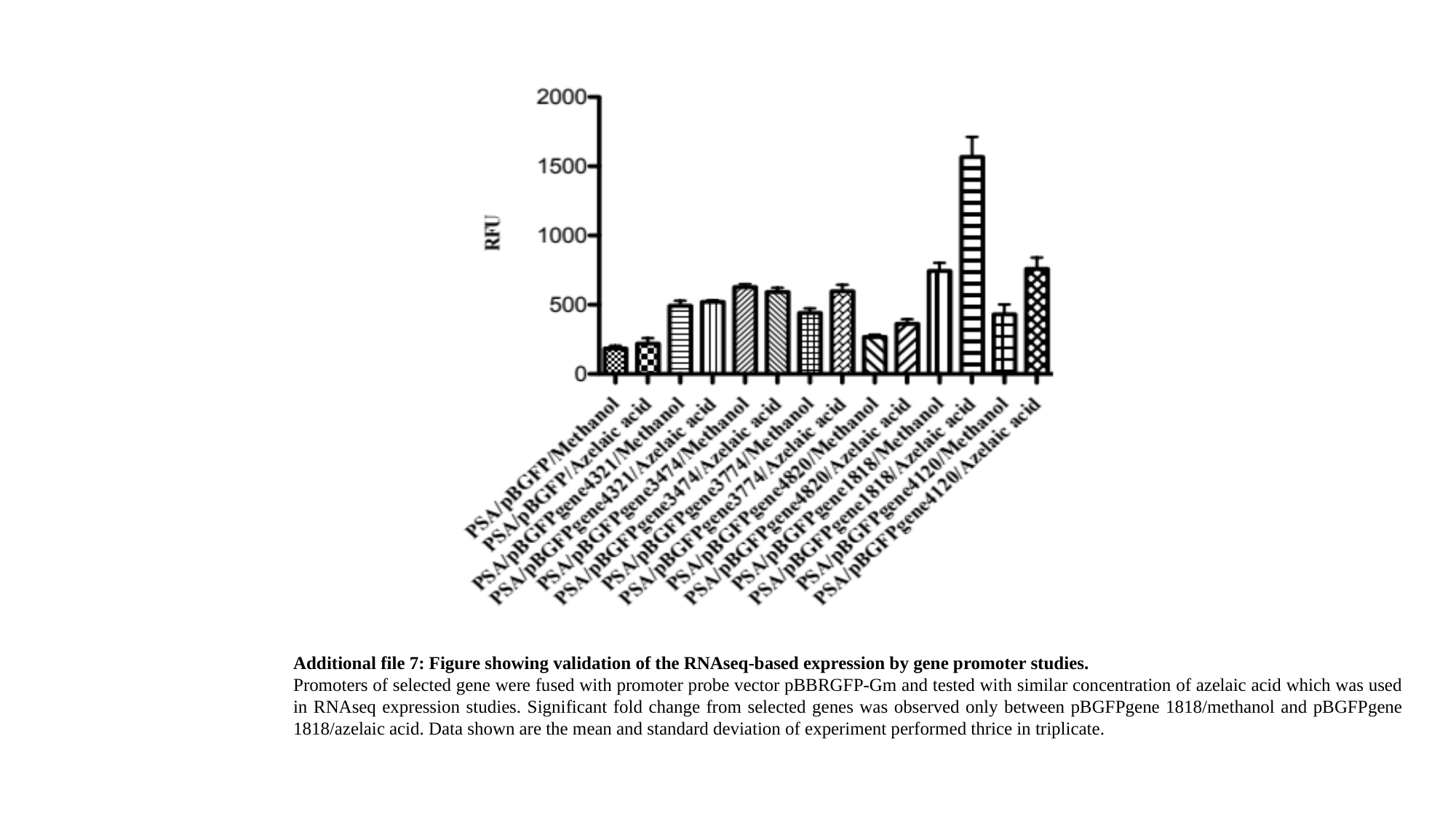

Additional file 7: Figure showing validation of the RNAseq-based expression by gene promoter studies.
Promoters of selected gene were fused with promoter probe vector pBBRGFP-Gm and tested with similar concentration of azelaic acid which was used in RNAseq expression studies. Significant fold change from selected genes was observed only between pBGFPgene 1818/methanol and pBGFPgene 1818/azelaic acid. Data shown are the mean and standard deviation of experiment performed thrice in triplicate.
